# Effect of Video Observation and Motor Imagery on Simple Reaction Time in Cadet Pilots

**DOI:** 10.3390/jfmk5040089

**Published:** 2020-12-05

**Authors:** Felice Sirico, Veronica Romano, Anna Maria Sacco, Immacolata Belviso, Vittoria Didonna, Daria Nurzynska, Clotilde Castaldo, Stefano Palermi, Giuseppe Sannino, Elisabetta Della Valle, Stefania Montagnani, Franca Di Meglio

**Affiliations:** 1 Department of Public Health, University of Naples “Federico II”, 80131 Naples, Italy; veronica.romano@unina.it (V.R.); annamaria.sacco@unina.it (A.M.S.); immacolata.belviso@unina.it (I.B.); dariaanna.nurzynska@unina.it (D.N.); clotilde.castaldo@unina.it (C.C.); stefanopalermi8@gmail.com (S.P.); giuseppe.sannino88@gmail.com (G.S.); elisabetta.dellavalle@unina.it (E.D.V.); montagna@unina.it (S.M.); franca.dimeglio@unina.it (F.D.M.); 2 Italian Air Force Academy, 80078 Pozzuoli, Italy; viria92did@gmail.com

**Keywords:** reaction time, pilots, motor imagery, video observation

## Abstract

Neuromotor training can improve motor performance in athletes and patients. However, few data are available about their effect on reaction time (RT). We investigated the influence of video observation/motor imagery (VO/MI) on simple RT to visual and auditory stimuli. The experimental group comprised 21 cadets who performed VO/MI training over 4 weeks. Nineteen cadets completed a sham intervention as control. The main outcome measure was RT to auditory and visual stimuli for the upper and lower limbs. The RT to auditory stimuli improved significantly post-intervention in both groups (control vs. experimental mean change for upper limbs: −40 ms vs. −40 ms, *p* = 0.0008; for lower limbs: −50 ms vs. −30 ms, *p* = 0.0174). A trend towards reduced RT to visual stimuli was observed (for upper limbs: −30 ms vs. −20 ms, *p* = 0.0876; for lower limbs: −30 ms vs. −20 ms, *p* = 0.0675). The interaction term was not significant. Only the specific VO/MI training produced a linear correlation between the improvement in the RT to auditory and visual stimuli for the upper (*r* = 0.703) and lower limbs (*r* = 0.473). In conclusion, VO/MI training does not improve RT when compared to control, but it may be useful in individuals who need to simultaneously develop a fast response to different types of stimuli.

## 1. Introduction

The time to respond to an external stimulus (reaction time) is the time lapse between the presentation of a stimulus and the onset of a voluntary response in a subject. The reaction time can be defined as the interval required to perceive the stimulus, process the information, fulfill an appropriate decision-making process, and initiate a motor task as a response [1]. Such a sequence of events adding to the reaction time is typical of real-life tasks and plays a critical role in many human activities related to sport or the professional performance of drivers, military personnel, security guards, or pilots. In neurophysiology, reaction time represents a valid indicator of an individual’s sensorimotor coordination and performance [2]. Three different types of reaction time can be described, based on the relationship between stimulus and response: simple, recognition, and choice reaction time [3]. In simple reaction time studies, there is one stimulus (auditory, visual or tactile) and one response. In recognition reaction time studies, stimuli to be responded to are interspersed with distracters that should not be followed with a response. In choice reaction time studies, several stimuli require different responses.

Reduction of reaction time is a desirable aim of intervention, both in the general population and its subsets, including athletes (e.g., swimmers or sprinters starting off the block in response to auditory stimulus or volleyball players pushing off in response to visual stimulus), patients affected by diabetes or osteoporosis (e.g., for fall prevention) [4], youths with intellectual disabilities, and patients with acoustic or visual impairment [5,6].

The reaction times of aviation pilots to auditory or visual stimuli and the skills in the execution of complex movements in response to these stimuli are of paramount importance during flight [7]. Aviation requires a combination of decision-making and kinesthetic skills. Many tasks during aircraft flying require continuous visual and auditory monitoring of the cues outside and inside the aircraft. Hence, the basic requirements of the pilot profession include fast and efficient information processing and fast and accurate reaction time [8]. Kennedy et al. [9] found that greater intra-individual variability in reaction time had an adverse impact on the ability of the pilot to maintain control of the aircraft in a flight simulator. These observations highlight the importance of the reduction of reaction time to different types of stimuli for the multitude of different tasks.

The motor imagery (MI) technique, trying to develop precise mental representations of the motor ability, led to improved performance of skilled movements [10]. Similarly, the video observation (VO) aids short-term motor skills learning [11]. These results can have the mirror neurons system as neurofunctional and neuroanatomical basis, a system able to facilitate subsequent movement executions by directly matching the observed action to the internal simulation of that action [12].

While it is known that MI and VO can exert positive effects on motor skill performance [13], few data are available regarding the effectiveness of these neuromotor training techniques in the reduction of the reaction times. Therefore, the aim of the present study was to investigate the influence of MI and VO on the simple reaction time to auditory and visual stimuli.

## 2. Materials and Methods

### 2.1. Subjects

The study protocol was approved in advance by the Ethical Committee of the University of Naples Federico II (22 March 2017, protocol number 58/17). Each subject provided written informed consent before participating. Participants were recruited on a voluntary basis among adult (age >18 years) male pilot cadets enrolled at the Italian Air Force Academy in Pozzuoli (Italy). Subjects with painful conditions during the three previous months and subjects affected by known orthopedic, rheumatologic, visual, acoustic, or neurological diseases that could interfere with the correct execution of the study protocol were excluded.

### 2.2. Procedure

Before randomization, all eligible subjects performed a pre-test evaluation of the imagery ability, using the revised movement imagery questionnaire (MIQ-R) [14]. The MIQ-R scores were collected at baseline and compared between groups to test for the homogeneity in imagery ability but were not considered in randomization or as an outcome measure.

The auditory and visual reaction times were measured using the Optojump device (Microgate, Bolzano, Italy), a previously validated and used tool for the measurement of reaction times [5,15,16,17]. This device is based on an infrared led technology and composed of transmitting and receiving parallel bars. To measure the reaction time for the lower limbs, the bars were positioned on the floor and the subject stood between the bars. The subject received the instruction to jump, lifting both feet off the floor, in response to an auditory (sound produced by the device) or a visual (appearance of a green ball on the device screen) stimulus. For the measurement of the upper-limb reaction time, the bars were positioned on a table with adjustable height. The subject stood in front of the table and the device height was set to allow positioning of both palms flat on the table between bars with full bilateral elbow extension and wrist extension. The subject received the instruction to lift both hands in response to auditory or visual stimulus. Each subject was allowed a single practice attempt to gain confidence with the equipment. All tests were supervised by a trained physician who was blinded to the group allocation.

The main outcome measure was the reaction time expressed in milliseconds (ms). Auditory and visual reaction times for lower and upper limbs were assessed during the same session, in the following order: three trials of auditory reaction time for lower limbs, three trials of visual reaction time for lower limbs, three trials of auditory reaction time for upper limbs, and three trials of visual reaction time for upper limbs. The mean value of each triplicate measurement was calculated for statistical analysis.

Subjects were randomly assigned to the control or experimental group, using dedicated online software (https://www.sealedenvelope.com/simple-randomiser/v1/lists). Following randomization, each subject received an identical-looking USB pen drive containing a video demonstrating the motor tasks necessary to complete either sham (control group) or specific (experimental group) VO/MI intervention protocol. All cadets participated in a training session dedicated to the principles and aim of the VO and MI techniques. Subjects were informed not to discuss the protocols, observe others during VO/MI protocol execution, or share any information about the study throughout its duration.

The VO/MI intervention comprised individual, supervised and non-directed sessions, carried out regularly for 4 weeks. For both sham VO/MI (control) and specific VO/MI (experimental) groups, each task represented in the video was repeated three times in a loop. No instructions were provided in the video about the observed tasks and their subsequent imaging and execution. The video, watched on a laptop 9.7″ screen, had no audio, except for the sound in the task related to the auditory stimulation reaction time assessment observed by the specific VO/MI group.

Each VO session was followed by an MI session and then the actual movement execution. All subjects were instructed to keep their eyes closed during MI. The whole routine, involving imagining the tasks observed in the video and actual movements, was performed by the cadets, always using the same equipment and at the same time of the day. Recorded tasks were performed by a male age-matched model wearing the leisure uniforms worn by all cadets at the Academy. Subjects allocated to the experimental group watched a video depicting the auditory and visual reaction time assessment, which was identical to that carried out during the baseline and end-point assessments. Hence, the experimental group performed an MI activity and then the actual movements based on VO aimed at improvement of auditory and visual reaction times. Subjects in the control group watched a video depicting activity included in an everyday physical training program (running, static bench, full push-up, standing toe touch), performed in the gym, followed by VO and MI procedures involving those activities. Thus, the VO/MI practiced by the control group was not related in any way to the end task for which the reaction time was measured.

### 2.3. Statistical Analysis

The main outcome of the study was to assess the change in the reaction time to auditory and visual stimulation following a neuromotor intervention comprising specific VO/MI compared with a sham neuromotor intervention. Therefore, the null hypothesis of the study was that the specific VO/MI would have no impact on the reaction times.

The distribution of continuous variables was assessed using the Shapiro–Wilk test and reported as mean ± SD. The MIQ-R scores were considered ordinal and reported as median and interquartile range (IQR) for visual and kinesthetic subscales. Data were analyzed by a Mixed Model ANOVA. Time was considered as within-subjects factor (baseline and post-intervention evaluation). Sham and specific interventions (group variable) were considered as the between-subjects factor. Interaction between time and group was investigated. The correlation between the Δ Reaction Time to auditory and the Δ Reaction Time to visual stimulus was assessed by Pearson correlation coefficients. All tests were considered significant if the p value was less than 0.05. Data analysis was performed using STATA software (StataCorp. v.12, College Station, TX, USA).

## 3. Results

In total, 41 males were assessed for eligibility. One cadet was excluded due to a recent orthopedic injury. Included subjects were randomly assigned to the control (*n* = 19) or intervention (*n* = 21) group. The mean age was 21.05 years (SD 0.97, range 20–23) in the control group and 20.7 years (SD 0.96, range 20–23) in the experimental group (*p* = 0.573). The MIQ-R scores were similar between groups (control group: median 20, IQR 19–21; experimental group: median 19, IQR 18–20, *p* = 0.105 for the kinesthetic subscale and control: median 19, IQR 18–19; experimental: median 17, IQR 17–19, *p* = 0.101 for the visual subscale).

**Figure 1:**
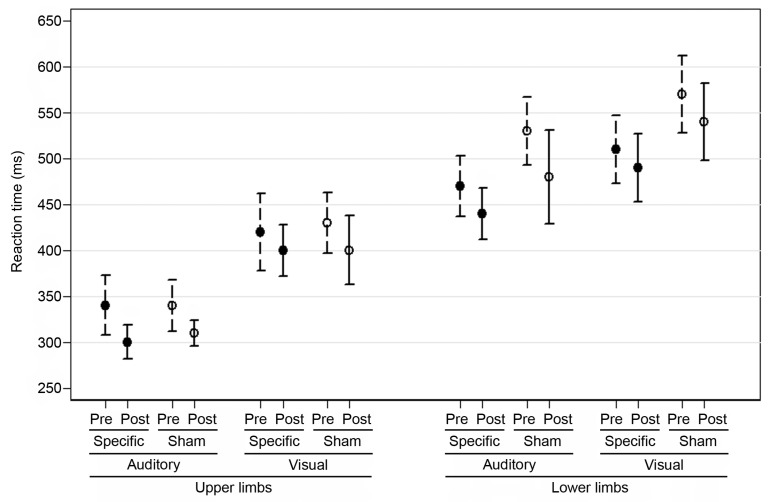
Reaction times to visual and auditory stimuli for upper and lower limbs in the experimental (specific VO/MI) and control (sham VO/MI) groups. Data are reported in milliseconds as a mean and 95% confidence interval. Solid circle indicates experimental group (specific intervention), hollow circle indicates control group (sham intervention).

The mean scores for the reaction time to auditory and visual stimuli for the upper and lower limbs pre- and post-intervention are reported in Figure 1 and the results of the analysis are reported in Table 1. The reaction time to auditory stimuli for the upper and lower limbs post-intervention improved significantly in both groups (control: mean change −40 ms, SD 40, experimental: mean change −40 ms, SD 80 for upper limbs; control: mean change −50 ms, SD 140, experimental: mean change −30 ms, SD 70 for lower limbs). A trend towards reduced reaction time to visual stimuli for upper and lower limbs was also observed in both groups (control: mean change −30 ms, SD 90, experimental: mean change−20 ms, SD 100 for upper limbs; control: mean change −30 ms, SD 90, experimental: mean change −20 ms, SD 80 for lower limbs). The effect of time was significant in all groups. The group effect for auditory and visual RT was not significant in upper limbs, while it was significant in lower limbs. In all comparisons, interaction term was not significant.

**Figure 2:**
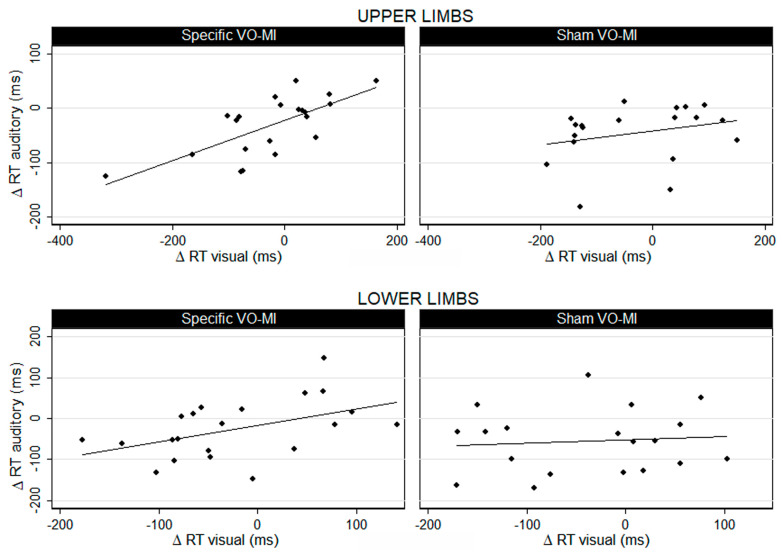
Correlation between the reduction in the reaction time (ΔRT) to visual and auditory stimuli for upper and lower limbs in the experimental (specific VO/MI) and control (sham VO/MI) groups.

While results showed similar improvement in the reaction times in both groups, the correlation between the reduction of the reaction times to visual and auditory stimuli differed between the groups (Figure 2). In the experimental group, reductions in the reaction times to visual and auditory stimuli were significantly correlated, with a high coefficient, for both upper (*r* = 0.703) and lower limbs (*r* = 0.473). Conversely, this correlation was not significant in the control group (*r* = 0.262 for the upper and *r* = 0.09 for the lower limbs).

## 4. Discussion

Our study demonstrated that a neuromotor intervention comprising a specific VO/MI did not significantly improve reaction times to visual and auditory stimuli for upper or lower limbs when compared with controls. However, the correlation between the reduction in reaction time to visual and auditory stimuli was demonstrated only in the experimental group. Importantly, we observed that the reaction times for upper and lower limbs to auditory stimuli in both groups were lower than those registered for visual stimuli at baseline. Following the specific or sham VO/MI training, reductions in reaction times in the experimental and control groups were always more significant for the auditory than visual stimuli.

Specific MI/VO sessions were programmed to respect the elements of successful interventions identified by Schuster et al. [18]; these were individual, supervised and non-directed sessions, added after physical practice. Furthermore, the whole specific routine (VO followed by MI followed by movement execution) was based on the approach suggested by Holmes and Collins [19], which incorporates physical, environment, timing, task, learning, emotion, and perspective (PETTLEP) elements into imagery. Monitoring and adjusting for as many PETTLEP elements as feasible were recently proposed to optimize the intervention outcome and to maximize the functional equivalence of imaged and actual task execution, since the PETTLEP technique was associated with a greater ease and/or vividness of the MI [20]. Its effectiveness was observed in several fields where the best possible performance of movement is crucial, such as sport, surgery or music [21]. Despite those previously reported positive effects in the performance of the movements, in our study, the VO/MI technique incorporating PETTLEP elements showed no advantage in improving reaction times to auditory and visual stimuli over the control group, in which those principles were not respected. This is in contrast with findings published by Simons et al. [22], who found that brain-training interventions improve performance in the trained tasks but not in the unrelated tasks. Nevertheless, their literature review did not include studies of MI or VO but focused on studies aimed at improving cognitive skills rather than simple reaction times.

Knowing that the reaction times positively correlate with physical fitness level [23], it can be argued that mandatory participation in the physical training program, that is included in the schedule of all Air Force Academy students, could have contributed to the improved reaction times observed in both control and intervention groups. Indeed, it was observed that students who exercised regularly had shorter reaction times than those who lead a sedentary life [24]. Shorter simple reaction times were also observed in elderly diabetic patients with or without neuropathy after moderate or intense supervised exercise program compared with pre-training [25]. Similarly, reaction time improved in children and adolescents with mild intellectual disability who participated in physical fitness training programs, compared to a control group [26]. Recent studies suggest that the negative effects of stressful conditions on workplace performance and, presumably, reaction time could be overcome by physical training [27,28].

The reaction time represents a complex neuromotor skill and it can be influenced by several external and internal factors, including type of stimulus (auditory, visual, or tactile), sex, age, physical fitness, level of fatigue, distraction, alcohol, personality type, dominant limb, biological rhythm, and health [13,23,29,30]. Accordingly, the study population selected for our study was homogeneous by gender, age, instruction level, biological rhythm, professional demands, instruction, and physical fitness, owing to a common resident training program applied to the entire sample by the Air Force Academy. Although it was not possible to control for all variables able to influence reaction times, many of the considered factors were homogeneously distributed in the study population.

Admittedly, the present study has still some limitations related to the subject of the investigation. Because studies of VO/MI effects on reaction time are lacking, we calculated the sample size for our study based on similar data on motor performance published elsewhere [13] and assumed a reduction of 150 ms in the auditory and visual reaction time as clinically significant. The absence of significant differences between groups could be caused by a high type II error with a low power of the results, due to the limited sample. As stated above, our study sample is represented by a highly homogenous population of the same gender, age, social status, lifestyle, education and physical activity. While this consideration allowed us to achieve an internal validity of the study design and results, at the same time, it may limit the generalization of the results. Regarding the choice of intervention, recent data suggested that a combination of model observation and self-observation had better short-term effects on motor performance than each VO method applied separately [11]. Nevertheless, only ideal model observation was used in the present study to explore, for the first time, its effect on the simple reaction time in association with MI. Further studies may be required to evaluate if the combination of both VO variants is more beneficial. Additional uncontrolled factors influencing the results of our study are placebo and expectation effects. Our choice of sham intervention in the control group, in which the VO/MI procedure was followed, but it was not related to the end task for which the reaction time was measured, allowed us to create an active control group. Recent studies indicate, however, that it may not be sufficient for eliminating a placebo effect, since the experimental and active control group can develop different expectations of improvement, even if interventions are incomparable [31].

In activities that take place in relatively unpredictable and constantly changing environment, movements have to be continuously adapted. Thus, developing physical and motor capabilities is as important as improving sensory and cognitive skills. This is particularly relevant for, but not limited to, open-skill sport activities [32]. In closed-skill sports (swimming, running), reaction time to auditory stimulus is often determinant for success. Additionally, in some professional activities, such as aviation, reaction must follow prompts of different types, including visual and auditory stimuli. The current study found that specific VO/MI training related to the end task, whereby the reaction time is measured, allows a parallel reduction in the reaction time for both types of stimuli. Further research in the cognitive and neurophysiological field, possibly incorporating the concept of spatiotemporal window for multisensory integration [33], is needed to explore how this correlation influences the information process; this could lead not only to better simple reaction time, but also to better recognition or choice reaction times.

## 5. Conclusions

In conclusion, we observed that the reaction times for upper and lower limbs to auditory stimuli were always lower, at baseline, than those registered for visual stimuli. The neuromotor training comprising specific VO and MI procedures in the experimental group did not determine a significantly higher reduction in the simple reaction time to auditory and visual stimuli than the sham procedure in the control group. Indeed, a significant reduction of reaction time to auditory stimulus and a trend towards reduction of reaction time to visual stimulus was observed post-intervention for the upper and lower limbs in both groups. Only the specific VO/MI training, however, produced a linear correlation between the improvement in the reaction time to auditory and visual stimuli. Interestingly, the reductions in reaction times in the experimental and control groups were always more significant for the auditory than visual stimuli. These findings could be crucial in training programs for aviation cadets and other professionals who need to improve their reaction times to a multitude of stimuli.

## Figures and Tables

**Table 1:** Reaction times (RT) to auditory and visual stimuli for the upper and lower limbs pre- and post- VO/MI training in the control and experimental groups.

Limbs RT in ms	Control Group			Experimental Group		
	Pre, Mean (SD)	Post, Mean (SD)	Change, Mean (SD)	Pre, Mean (SD)	Post, Mean (SD)	Change, Mean (SD)
Auditory, upper	340 (60)	310 (30)	−40 (40)	340 (70)	300 (40)	−40 (80)
Visual, upper	430 (70)	400 (80)	−30 (90)	420 (90)	400 (60)	−20 (100)
Auditory, lower	530 (80)	480 (110)	−50 (140)	470 (70)	440 (60)	−30 (70)
Visual, lower	570 (90)	540 (90)	−30 (90)	510 (80)	490 (80)	−20 (80)
